# Exploring Patient Participation in AI-Supported Health Care: Qualitative Study

**DOI:** 10.2196/50781

**Published:** 2025-05-05

**Authors:** Laura Arbelaez Ossa, Michael Rost, Nathalie Bont, Giorgia Lorenzini, David Shaw, Bernice Simone Elger

**Affiliations:** 1 Institute for Biomedical Ethics University of Basel Basel Switzerland; 2 Care and Public Health Research Institute Maastricht University Maastricht The Netherlands; 3 Center for Legal Medicine (CURML) University of Geneva Geneva Switzerland

**Keywords:** artificial intelligence, AI, patients, qualitative research, patient empowerment, shared decision-making, AI-driven care, AI-support, AI ethics, responsible AI, patient participation

## Abstract

**Background:**

The introduction of artificial intelligence (AI) into health care has sparked discussions about its potential impact. Patients, as key stakeholders, will be at the forefront of interacting with and being impacted by AI. Given the ethical importance of patient-centered health care, patients must navigate how they engage with AI. However, integrating AI into clinical practice brings potential challenges, particularly in shared decision-making and ensuring patients remain active participants in their care. Whether AI-supported interventions empower or undermine patient participation depends largely on how these technologies are envisioned and integrated into practice.

**Objective:**

This study explores how patients and medical AI professionals perceive the patient’s role and the factors shaping participation in AI-supported care.

**Methods:**

We conducted qualitative semistructured interviews with 21 patients and 21 medical AI professionals from different disciplinary backgrounds. Data were analyzed using reflexive thematic analysis. We identified 3 themes to describe how patients and professionals describe factors that shape participation in AI-supported care.

**Results:**

The first theme explored the vision of AI as an unavoidable and potentially harmful force of change in health care. The second theme highlights how patients perceive limitations in their capabilities that may prevent them from meaningfully participating in AI-supported care. The third theme describes patients’ adaptive responses, such as relying on experts or making value judgments leading to acceptance or rejection of AI-supported care.

**Conclusions:**

Both external and internal preconceptions influence how patients and medical AI professionals perceive patient participation. Patients often internalize AI’s complexity and inevitability as an obstacle to their active participation, leading them to feel they have little influence over its development. While some patients rely on doctors or see AI as something to accept or reject, these strategies risk placing them in a disempowering role as passive recipients of care. Without adequate education on their rights and possibilities, these responses may not be enough to position patients at the center of their care.

## Introduction

### Background

There is significant enthusiasm about the potential applications of artificial intelligence (AI) in health care [1]. In particular, the AI subtechniques of machine learning (ML) could, in the foreseeable future, be widely implemented to support triage, diagnosis, and treatment—especially in the form of clinical decision support systems (CDSS) [2,3]. With AI’s implementation, there are significant hopes that AI can alleviate an overwhelmed health care system by supporting doctors in their daily decisions and data analysis tasks. However, the medical AI community must navigate complex ethical, technical, and human-centered challenges to exploit these potential opportunities [4]. With the introduction of AI, health stakeholders will need to re-evaluate commonly held conceptions about what constitutes good health care. For example, there are unanswered questions about how the introduction of AI will affect the patient’s role in AI-supported health care and whether their preferences and expectations align with common ethical paradigms and practices such as shared decision-making (SDM).

Health care, is at a crossroads, grappling with the need to provide quality care while managing vast amounts of medical knowledge and data. Health data are now a part of care interactions, and analyzing it with AI is viewed as essential for improving health care outcomes by providing insights that support better clinical decisions [2]. While AI is a broad term, it is often defined by its behavior and functionality [5]. For example, the High-Level Expert Group on Artificial Intelligence of the European Commission defines AI as systems designed by humans to achieve complex goals by perceiving their environment, processing data, and deciding on actions to meet those goals [6]. Based on this definition, AI encompasses a wide range of technologies, from ML techniques to large language models such as ChatGPT or DeepSeek. Regardless of the underlying AI technique, the road ahead for AI CDSS is to identify whether AI can deliver enough value to match its expectations and whether it can do so within the limits of ethical behavior. Despite AI’s hype, a comprehensive exploration of this technology’s ethical implications and boundaries remains elusive, especially regarding patients’ preferences and how to involve AI in their care ethically.

Historical accounts showed that patients used to have a passive acceptance role where they mostly followed doctors' suggestions and did as they were told. Although now considered ethically unacceptable, past paternalistic models in health care limited patients' freedom of choice and participation in decision-making [7]. Understandably, new acceptable ethical models focus on person-centered care, enhancing patient participation, and supporting SDM. In SDM, a collaborative process between patients and health care professionals, it is essential for patients to be actively involved in their care [7,8]. In that sense, patient participation is an essential step of person-centered care as it focuses on involving patients in the decision-making process or other aspects of health care, including education, goal setting, self-monitoring, or taking part in medical examinations [9]. Person-centered care gives patients a central role by including them in the care process and adapting to their needs and expectations to support patient empowerment [10]. This empowerment considers the complexity of subjective experiences (eg, hopes, fears, and views) and emphasizes the importance of sustaining patients’ autonomy and self‐determination in their care [10]. The expectation is that empowering patients allows them to engage in their care and gives patients a sense of control over their health journey, making them an active central part of their care. Empowering patients can make them more satisfied with their care, have a deeper engagement in their health, adhere more easily to treatments, and better cope with the uncertainties common to the progression of chronic diseases [11,12]. As the World Health Organization stated in their framework for person-centered health care, there must be a shift towards health systems designed for people, coproduced for their needs and preferences, and consciously adopting the perspective of individuals, families, and communities [12]. Involving patients in their care and providing them with the resources and tools to promote active participation better equips them to understand their health journeys and ultimately make the decisions that align with their values and preferences [7].

A 2023 systematic review found that while most patients and many health professionals have a positive attitude toward AI in health care, concerns persist about whether it can genuinely enhance care quality and improve patient experiences [13]. The authors highlighted the importance of human agency in AI-enabled health care. While health care professionals emphasized their agency and focus on their important role in AI development and implementation, discussions rarely addressed the participation and role of patients in AI-supported care. Debates have largely focused on how AI-generated health information, such as self-monitoring tools, could empower patients. However, they often overlook whether AI can truly support patient empowerment and foster person-centered care [14].

Researchers have focused on investigating the factors that influence whether patients accept or reject the use of AI, with particular emphasis on their preferences regarding data sharing and privacy. Understanding these preferences is crucial, as they shape patient trust in AI-supported systems and determine their willingness to engage with AI in health care [15-19]. However, few research studies have focused on understanding the role (engagement and participation in decision-making) that patients would like to have in AI-supported care. In the United Kingdom, although patients saw AI positively, they reported that to be comfortable with AI use, there must be the intention to preserve their choices (regarding whether AI is [or not] used and the ability to contest AI’s care decisions) [20]. However, most patients expected doctors to retain final discretion over the care plans and act as guardians protecting them from potential harm [20]. Similar to previous findings, these results show that although patients want to be engaged, how actively they wish to participate in decision-making is variable and possibly self-contradictory [21,22]. Patients may face many obstacles to participating in SDM. For example, a lack of knowledge and low health literacy could affect patients’ confidence to make decisions and reduce their willingness to participate in decision-making [9]. As the preferences of patients are individual, variable, and contextually dependent, patients could either decide to take a leading role in SDM or not exercise their right to participation during AI-supported care [14].

There is a risk that AI could create new paternalistic structures where AI holds the power to indicate how to act in patient care without much consideration of patients’ preferences and needs. To an extent, patients’ acceptance of AI could depend on their general perception of AI, their characteristics (eg, age, interest, and gender), and their previous experiences in care [21,23]. If patients have experienced paternalistic decision-making during their care—where the doctor said what should be done—they may be more likely to accept (or even endorse) paternalistic care [21]. In that sense, people developing and using AI in health care should pay particular attention to avoid a blind acceptance of paternalistic AI implementations, which could disincentivize patients' participation and risk their autonomy and self-determination.

### Objective

The introduction of AI into clinical practice brings potential challenges, particularly in terms of patient participation and empowerment. Despite AI's promise to enhance health care, there are concerns about whether AI technologies can meet the ethical standards of medical practice [24-26]. Patients and professionals alike may acknowledge the risks associated with AI, particularly to autonomy and patients' and doctors’ decision passivity [27,28]. If patients are not properly educated about their rights and the possibilities that AI offers, they may feel disempowered, accepting AI as an inevitable part of their care without fully understanding its implications. This raises questions about the extent to which AI can risk patient empowerment and consequently, SDM. Therefore, when the goal is to maintain person-centered care and support patient empowerment, it is vital to understand the elements that define patients’ preferences concerning participation in decision-making during AI-supported care.

This research aims to explore the role patients prefer to take when AI is involved in their care, offering a critical analysis of the external and internal factors that may influence their level of participation in decision-making. Using a qualitative approach, we examined the perspectives of both patients and professionals working with medical AI (referred to as AI professionals). With a focus on person-centered care, this study aims to amplify the patient’s voice by acknowledging their experiences, perceptions, knowledge, attitudes, and beliefs that shape their preferred role and their envisioned interactions with AI in health care. The insights gained will encourage both patients and professionals to critically engage in discussions about patient preferences, and establish the boundaries and conditions needed to support patient empowerment in the context of AI-supported health care.

## Methods

### Qualitative Approach and Context

The qualitative approach for this study is semistructured interviews for data collection and reflexive thematic analysis (RTA) as our analysis framework. Semistructured interview guides were used for data collection, reflecting the study's exploratory approach and allowing for in-depth conversation with every participant. We used RTA as our analytical framework because it allowed us to situate the analysis in the context of health care interactions and determine in-depth and implicit patterns of meaning across the data [29]. According to Braun and Clarke [29], RTA is well-suited for research that requires a nuanced exploration of complex attitudes and beliefs—such as those surrounding AI in health care. With evolving technology such as AI, RTA allows to interpret the data in depth capturing both explicit content and underlying themes, such as ethical considerations or relational dynamics. As a postpositivism methodology, this method focuses on the depth and richness of the insights gained, contributing to a more comprehensive understanding of participants’ views [30]. This methodology enabled us to contextualize our analysis for health care and uncover intricate and underlying patterns of meaning within the available data [29]. This study follows the standards for reporting qualitative research (SRQR) [31].

The data for this manuscript is drawn from a larger research project titled “Ethical and Legal Issues of Mobile Health-Data: Improving Understanding and Explainability of Digital Transformation and Data Technologies Using Artificial Intelligence (EXPLaiN),” funded by the Swiss National Science Foundation. The research group acknowledged their positionality as researchers and how their ethical backgrounds, which make patient empowerment vital, informed the interpretation of these results. However, to prevent a single or superficial view, our research group engaged in frequent discussions and included different academic backgrounds (philosophy, ethics, medicine, and psychology).

### Participants

#### Recruitment

We recruited 2 subgroups of participants, patients, and AI professionals. To recruit patients, we approached patients seeking outpatient care at the University Hospital of Basel. Participants were purposely sampled to generate a diverse sample that included a mix of older and younger patients. Therefore, we recruited patients consulting the hospital for the cardiology and infectious diseases departments as they typically are a large outpatient group. Patients must be 18 years or older, consented to participate in 30- to 45-minute interviews in English, Swiss German, or German, and signed the consent form. There were no restrictions on patients based on disease history or diagnosis. Patients were contacted face-to-face at the hospital during their scheduled consultations. Researchers made weekly visits to the hospital, contacting on average 5 patients during each visit. During face-to-face recruitment, most rejections involved patients expressing a lack of interest, time constraints, or practical challenges like language limitations or hearing impairments.

AI professionals were selected purposefully from a range of disciplines, including medicine, bioethics, public health, philosophy, psychology, economics, law, and computer science. To be included, participants needed to have professional experience with medical AI and hold a senior position in either academia or the private sector, excluding PhD students, interns, and early-career professionals. Examples of participants' roles included professors at universities, senior managers of AI-focused companies, and senior data protection officers in hospitals. Participants were identified based on their involvement in medical AI through projects, products, or research, and were contacted via publicly available email addresses on institutional or corporate websites. A snowball sampling technique was also used, with participants invited to refer others who met the inclusion criteria. Using purposive sampling based on experience allowed us to produce rich, articulated, and expressive data [32]. The initial contact with participants was made via email, inviting them to take part in an interview. The email provided an introduction to the project, outlining its objectives and explaining what their participation would involve, including details on time commitment, audio recording, transcription methods, and the format used for data pseudonymization.

#### Sample

The first phase of the study involved 41 semistructured interviews with global participants exposed to medical AI, representing various disciplines including medicine, philosophy, law, ethics, public health, and computer science. These interviews explored the barriers and facilitators to implementing AI in clinical settings, particularly in relation to CDSSs and wearable devices. The second phase involved 21 semistructured interviews with patients assisting the University Hospital in Basel, Switzerland. The original study aimed to understand current perspectives, attitudes, knowledge, and challenges regarding AI's role in health data analysis and its potential to support decision-making for both patients and AI professionals. This analysis focuses on a subset of the data collected on AI professionals and patients located in Switzerland or Germany. The goal was to create geographically comparable groups to the patients that lived in the German-speaking part of Switzerland in Basel close to the border with Germany. This translated into a selection of 50% of the AI professional sample and 100% of the patient sample.

### Ethical Considerations

The Ethics Committee of Northwest and Central Switzerland approved all methods and this study according to the Human Research ACT Art. 51 (approval number: AO_2021-00045). According to the Ethics Committee of Northwest and Central Switzerland, interviewing professionals were exempt from the Swiss Human Research ACT given no sensitive data was collected. Therefore, the professional interviews required no formal written consent and only needed verbal consent at the beginning of the interview. Patients' written consent was collected and stored locally at the University compounds as per ethical approval protocol. All personal data were pseudonymized and securely stored on the University of Basel's server, with access to the key restricted to the research team. Any potentially reidentifiable data were excluded from data analysis and publications. Participants did not receive any compensation.

### Data Collection

Three members of the research team (LAO, NB, and GL) recruited participants and conducted one-on-one semistructured interviews. LAO completed 11 patient interviews. After training with LAO, NB conducted 10 patient interviews in Swiss German, which allowed patients to communicate more comfortably in their native language. Patient interviews, held at the hospital, were conducted in either English or German and recorded with an audio device. GL led 7 and LAO 13 interviews with AI professionals, recorded via Zoom and stored locally. All interviews were conducted between March 2021 and May 2023. LAO and GL transcribed the English interviews verbatim from AI professionals and patients, while NB transcribed those conducted in Swiss German or standard German.

The research team designed interview guides for each subgroup, focusing on key areas: general impressions of AI, the AI-patient relationship, and the AI-doctor-patient relationship. Although the interview guides were largely similar, the version for AI professionals included additional in-depth questions, exploring topics such as interpretations of AI regulations and specific AI concepts. Vignettes were used during both sets of interviews as a tool to enhance discussion among the participants. Using vignettes which are brief descriptions of scenarios, enables researchers to explore participants' attitudes and beliefs without requiring extensive familiarity with the research topic. This approach is especially beneficial for studies on technology applications, as vignettes provide theoretical advantages when exploring abstract or complex topics that may be challenging to discuss directly [33,34]. This is especially evident in medical AI where there are potential implementation scenarios but still limited real-world experiences. The questions were organized into 6 sections: introductory general questions on AI use in medical practice, context-specific questions on AI-patient relationships (using vignette 1 with a wearable device), context-specific questions on doctor-patient relationships involving AI (using vignette 2 with CDSS), and concluding questions. While we acknowledge that responses to vignettes are often shaped more by personal views and moral intuitions than by participants’ theoretical knowledge, the way participants interpret these scenarios reflects how they navigate and make sense of their daily lives [34]. The vignette approach benefits qualitative studies by eliciting authentic responses that mirror real-life reasoning and allow participants to project personal values and intuitions onto scenarios, offering deeper insights into their attitudes and beliefs, and enhancing the richness of the data collected [33,34]. The interview guides are available as Multimedia Appendix 1.

### Data Analysis

The authors (LAO, MR, and NB) led the analysis, and all the coauthors supported the analytical process. We carried out inductive and deductive thematic coding of the data, initially line by line, using descriptive or latent labels (MAXQDA software; VERBI Software). AI professionals' and patients’ interviews were coded initially separately. LAO and GL coded all the AI professionals’ interviews. LAO and NB coded patient interviews. Researchers merged AI professionals' and patients’ data and their initial codes. The authors (LAO, MR, and NB) developed overarching themes and subthemes to identify commonalities across the data, which were then reviewed by the entire research team. After recurring discussions and refinements, the team created 3 major themes illustrating how patients and AI professionals envision patients' role in AI. All interviews were analyzed in the original language (German/English). The researchers' backgrounds played a key role in shaping the interpretation of the data, leading to the development of themes that highlight often-overlooked ethical concerns, particularly the challenges to patient participation and empowerment. We focus on questions related to, person-centrism, a widely acknowledged paradigm that helps to question and reflect on power structures and how these affect patients. While our positionality influenced the analytical process, we actively tried to reduce biases through ongoing discussions within a multidisciplinary research team, incorporating expertise from philosophy, ethics, medicine, and psychology. This collaborative approach ensured a more nuanced and comprehensive analysis.

We aimed to identify areas of consensus where patients and AI professionals shared views, fears, and beliefs about AI in health care. To illustrate the presented findings, we used representative (disidentified) extracts. The patient participant (PT) acronym recognizes patients' quotes, and the AI professional participant (AE) recognizes AI professionals. To improve readability, the authors removed filler sounds and double words from the data presented in this paper. For this publication, the authors translated the extracts as required (LAO, NB, and MR).

## Results

### Overview

The study included 2 subgroups: 21 patients and 21 AI professionals for a total of 42 interviews. A couple was interviewed together for the patient group (PT9.1 and PT9.2). Given that AI in health care is a multidisciplinary area, most professionals found themselves at the intersection of 2 or more areas of experience; for example, 8 participants were doctors with AI experience. Patient participants came from diverse educational backgrounds and were all recruited in Basel, Switzerland. AI professionals represented fields such as medicine, sociology, law, and computer science, with 13 based in Switzerland and 8 in Germany. The gender distribution was similar in both groups, with 7 women and 14 men among patients, and 6 women and 15 men among AI professionals. Detailed sample characteristics are available in Multimedia Appendix 2.

**Figure 1:**
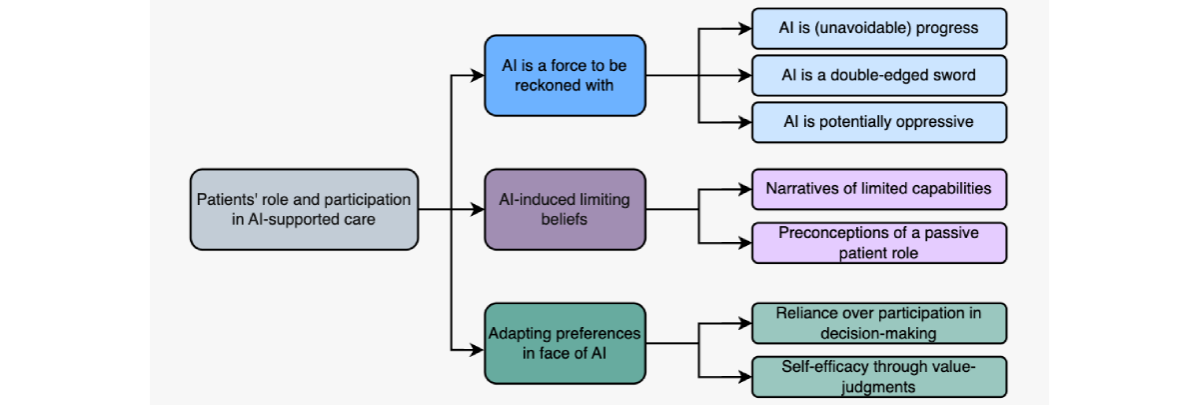
Map of themes illustrating the views on the patients' role and participation. AI: artificial intelligence.

The results of this research are structured around 3 key themes. The first theme explores the perception of AI as an inevitable and potentially disruptive force in health care, reflecting both optimism and concerns about its transformative impact. The second theme examines how patients perceive limitations in their own capabilities, which may hinder their ability to actively participate in AI-supported care. This includes uncertainties about how much patients can influence AI systems, concerns about decision-making authority, and fears of losing personal agency. The third theme describes patients’ adaptive responses to AI integration, such as relying on expert guidance or making personal value judgments that lead to either acceptance or rejection of AI-supported care. These themes collectively illustrate the complex and often conflicting ways in which patients navigate the presence of AI in health care settings (Figure 1). To strengthen the results, key ideas will be supported by participant statements, with identifiers (eg, [PT8]) indicating who expressed each idea. However, this does not mean these participants were the only ones to share this perspective; rather, these examples were chosen as particularly clear or representative illustrations of themes. Full quotes can be found in the theme tables for context.

### AI Is a Force to Be Reckoned With

#### Overview

This theme explores the external influences (eg, narratives, myths, science fiction, and fears) that shape the perspectives of patients and professionals regarding AI in health care (Table 1). Either positively or negatively, participants considered AI as a forceful driver of change. Most patients and some professionals determined that AI is an unavoidable future or thought AI to represent primarily positive progress. Some patients described AI as a double-edged sword that depends on humans' use. At the same time, some professionals discussed this risk regarding the involvement of private companies in AI development. In particular, patients expressed concerns that AI may have an oppressive potential as it could excessively monitor their health or exert control over their lives and health decisions. All these external accounts influence the expectations of a future with AI for patients and professionals and the participatory preferences of patients.

**Table 1:** Data extracts representative of the theme “AI is a force to be reckoned with.”

Subtheme and participant		Data extract
**AI^a^ is unavoidable**		
	[PT1]^b^	*Because medical progress. As simple as that. So, why should I use something which has less diagnostic or therapeutic powers when something better is available? (...) technology is an improvement to the old times. Isn't it?.*
	[PT5]	*I cannot change it anymore. It is simply part of the present age with digitization, one cannot stop it.*
	[PT10]	*Yes, we have no other choice left. Because in 10 years you have no more doctors and afterward you should be able to treat more people with fewer people. And, that is only possible by automating and combining certain things. It is a must.(...) And there we need computers as support, which then tells you, it narrows the whole thing down and then the doctor has to come. But otherwise we will no longer make it.*
	[AE12]^c^	*I guess you cannot avoid it. It's already here. (...) So, it's not, I don't think the question is to be in favor or against machine learning or whatever. (...). It's just here, and now we need to deal with it.*
**AI is a double-edged sword**		
	[PT19]	*So I think it [AI] will come. I am quite sure that this will come. And as long as it comes to the fact that humans, animals, the environment could benefit from it, I am very much for it.*
	[PT8]	*I think it [AI] has opportunities and risks; it is always, with everything the question of who does what with it. I think you can use it for many positive things, and you can also abuse it a lot. So, I think, maybe I hope, that it is more of a positive one. But it is through all the technical things, I can use everything in one or the other way. I can use a drone to take beautiful pictures and deliver parcels, or I can use it somehow to wage war somehow, and this will potentially be done with an AI.*
	[AE16]	*The other is that you have young people, they do startups, they are idealistic people, and they are trustable people, but they are naive people. And they don't weigh properly the good their system could do, and there is no doubt that these systems can do good, but they don't measure the bad, the pain these systems can do. They understand that it is important not to miss a breast cancer in a woman. They don't understand the shock and the energy, and the pain that the fear of a breast cancer can induce in a person for months because you have something that said you have a risk. And for these people, from one second to the other, the sun is away, the day becomes night, and every minute of their life becomes anxious. And they don't measure the costs, not the money, but the human cost of false positives.*
	[AE13]	*There are going to be people who use it for good application and people who use it to exploit, right? And that’s just the way the world is, right? So, I don’t think anything is inherently good or bad, but it’s how something is then utilized. And there will be for sure bad actors and bad players.*
**AI is potentially oppressive**		
	[PT15]	*What will be, so it is the question, whether you really have to know that in detail now [their health data] and then give warnings. Life is vulnerable and that can end at some point and so on. Whether you don't question everything a little too much now and have everything ready and so on. I'm a little against it.*
	[AE4]	*I think it's very, yes, science-fiction based, irrational fear. But yeah, I mean we tend to be fearful of many things and in general we overreact to fear. So, in very strange circumstances we are kind of risk-takers, but most of the time we tend to be overly protective and overly restrictive. Because we overestimate these kinds of risks, due to misinformation, due to specific narratives that are created, specific images that are present. It's sufficient for this kind of fear to just have one story, you know almost, one urban legend that keeps returning, and people just kind of attached to that and yeah basically stop thinking about this in a rational manner.*

^a^AI: artificial intelligence.

^b^PT: patient participant.

^c^AE: AI professional participant.

#### AI Is (Unavoidable) Progress

Most patients and some professionals saw AI as representing unavoidable progress. From a positive perspective, AI is desirable and a token of medical progress as described explicitly by one participant (PT1). Although patients had a generally positive outlook on the progress brought by AI, they also felt a lack of empowerment. To an extent, patients thought that they could not influence the development of AI (PT5). Others described that AI could not be changed or stopped as it is already used in other systems for daily tasks (eg, search engines and bank systems; PT10). To justify their view that AI is inevitable, patients and professionals focused on the potential benefits or the faculties AI has to overcome human limitations. In particular, one patient (PT14) felt powerless against the desire to increase (process) efficiencies, making AI an incontestable development.

#### AI Is a Double-Edged Sword

The belief that AI constitutes unavoidable progress has prompted questions regarding its application and AI’s potential double-edged nature. Some patients reflected on the conditions for positive progress and required AI to be beneficial to accept it, for example; either beneficial for themselves or everyone (PT19). However, depending on the use, some patients fear AI may be exploited to benefit private companies or the hospital system instead of patients (PT8). Some professionals referred to the challenges of having private companies participating in health care as they might not understand the health care system or might look to take advantage of patients’ data for their interests (AE16 and AE13). Patients felt a lack of control and protection regarding the intentions of the people using or developing AI. In their view, technology depends more on who determines its goal than its technical characteristics. In that way, the double-edged sword potential of AI expressed a lingering fear of (other) people’s goals and not AI in itself.

#### AI Is Potentially Oppressive

In contrast to the use-dependent interpretation of a double-edged AI, several patients considered that AI could become an overlord in their lives. Concerning AI notifying them directly of health-related information (ie, changes in heart rhythm via a smartwatch), patients felt irritated or stressed (PT15). To some patients and professionals, this flow of information, often thought to empower patients, is undesirable and almost felt outside their control. In contrast to patients, professionals did not often mention the fear of an oppressive AI. Two professionals explicitly expressed that basing AI judgments on works of fiction could create dystopian fears among patients (AE4). In some respects, patients felt a tension between a potentially oppressive AI that might also be unavoidable and a desire to remain in charge of their own lives and health. Some patients explicitly agreed and expressed their views on science fiction tales of AI oppression. For example, 2 patients (PT3 and PT18) who expressed their desire not to be controlled by AI also mentioned how their first encounter with the topic was science fiction movies.

### AI-Induced Limiting Beliefs

#### Overview

The external view that AI is a force for change appeared to have influenced the (self) evaluation of patients’ capacities to handle the changes brought by AI (Table 2). Patients and professionals believe patients have limitations in engaging with AI. Both groups, in one way or the other, have accepted these limitations and put patients in a passive role where health care professionals need to shelter them from AI. These views will likely impact the expected role and preferences in the decision-making of patients, even if unintended.

**Table 2:** Data extracts representative of the theme “AI-induced limiting beliefs.”

Subtheme and participant		Data extract
**Narratives of limited capabilities**		
	[PT17]^a^	*I don’t feel intelligent enough to judge it [AI]. It exceeds my knowledge.*
	[PT6]	*You can slowly say at my age, 76, that there are things [when asked about AI] that you see from a certain distance. On the other hand, 10 or 20 years younger, you have to be there, that is very clear. But I am no longer in the work process, and financially, it is no longer a problem, so for certain things, someone can say I don't care.*
	[AE9]^b^	*Like, I mean, realistically you can say: “okay, there's a need to disclose a lot of things” [about AI] but of course, also patients can only meaningfully process so much information.*
**Preconceptions of a passive patient role**		
	[PT11]	*It's difficult, so I don't see it. Now as a patient, I come there and then something is offered to me, and then I can as a patient, what could I do differently myself.*
	[AE8]	*Patients are not, I don't think they are responsible, that they, they should know it [about AI] because they only just there to receive the treatments and, maybe, follow the advices of the doctors. But, I don't think they have the responsibility, and I would, don't give them the responsibility to understand the systems or to understand the recommendations or other outcomes. I would keep them out of this.*
	[PT7]	*Is it my right to say?[about how to support AI implementation]. Such questions I have been asked for the first time. I never thought about it. But yes, it is good to know that you want to know what patients think and listen to their opinion and let it play a part. I appreciate that.*

^a^PT: patient participant.

^b^AE: AI professional participant.

#### Narratives of Limited Capabilities

Several patients have internalized limitations regarding their roles and capabilities. Some patients felt AI is easy for young people or those considered experts (P17 and P6). In particular, patients reflected on their limits in understanding AI. Some patients further justified their limitations due to a lack of interest in technology or that they do not have to understand or relate to AI. In a way, AI was not for them but possibly for other (younger, smarter, and more interested) people.

Beyond their self-assessed lack of capabilities, some patients said that explanations provided by others might not help them feel more secure about their capacities, even if doctors provided these explanations. Like patients, several professionals questioned whether patients have the competencies to handle AI or if explanations could overcome the perceived limitations (AE9). Both groups accepted that AI is a complex subject and dismissed the opportunities for patients to acquire the skills to handle AI. However, many patients mentioned they actively use other forms of technology, such as smartphones or smartwatches.

#### Preconceptions of a Passive Patient Role

Beyond the perceived limited capabilities of patients to handle AI, patients position themselves in a passive (receiving) role. Several patients perceived that concerning AI, they would do as the doctor said because doctors are the experts or patients cannot do anything differently (PT11). A few professionals who determined that patients might not need to be involved in AI expressed the view of a passive patient (AE8). Protecting patients from the complexities and dangers of AI also meant excluding them from its development and use. This view implicitly removes the patients’ power to decide whether and how to participate in AI. Consequently, a few patients accepted their lack of participation as the standard of care, making them surprised or overwhelmed when questioned about their preferences and ideas regarding AI (PT7).

### Adapting Preferences in the Face of AI

#### Overview

Attributed and observed limitations may have influenced how patients and professionals perceive the patient's role in AI. These limitations have required patients to adapt—consciously or not—and find strategies to maintain their self-efficacy at least to a degree by handing over the responsibility for AI to their doctors. For patients, another way to regain decisional authority was to judge the value of AI for their lives and decide whether to accept or reject it depending on their preconceptions and their general technology-related values. These adaptive strategies influence what patients think is possible regarding their participation in AI and what they see as optimal and feasible (Table 3).

**Table 3:** Data extracts representative of the theme “Adapting preferences in face of AI.”

Subtheme and participant		Data extract
**Reliance over patient participation in decision-making**		
	[PT17]^a^	*In principle, I have more confidence in doctors, experts. And before I do not wish that the devices tell me “oh they have a problem here, there”, before experts get this information. You [the expert] will see how serious or how the situation is, they will sort and what is necessary you will tell me. This is the right process, I think.*
	[AE13]^b^	*This is the trust that you give a doctor that they do their job without you telling them what to do, right? So, it’s like, if a radiologist is looking for cancer or something, and they use a machine learning algorithm to better understand where, what could be like cancer cells or not, right? You don’t care, right? Maybe, it is different if you are, this is part of the doctor’s role and job, and if it is something like this where it is a tool that exclusively doctors use, I don’t really think there has to be much communication to the person unless if they are curious and ask.*
	[AE12]	*Probably the less you know, the more you trust. (...) And then, if you know too much about this [AI], the technical aspects, maybe you don't trust it anymore. (...) So, you can imagine anything, you know, depending on your knowledge.*
**Self-efficacy through value judgments**		
	[PT11]	*No. Because I have my computer and otherwise enough electronic gadgets. I don't need [AI-enabled smartwatches], no. I can imagine that [other people use them], but I do, well, I have somehow my attitude to life that I want to concentrate on myself and don't want to be distracted as much.*
	[PT6]	*I am not against it [about AI]. And other people that advocate against it, I mean this was crazy during the pandemic how those two disparities (…) it is mere ideology.*
	[PT5]	*And that's exactly how it is, actually. Either I say, I trust in the technology that it does it correctly, that it is checked, that the result is correct. Or I reject it and say “no, I can't imagine that”. But that is again the personal feeling of the patient, where he says, “yes, I could imagine that now”, the result or “no, I don't want that”.*

^a^PT: patient participant.

^b^AE: AI professional participant.

#### Reliance Over Patient Participation in Decision-Making

The notion of a passive role is ingrained in patients’ beliefs that those providing care know what is best for them and could potentially make better decisions than themselves. Therefore, patients chose to rely on doctors to protect themselves from the distress of using AI, mainly as they felt limitations regarding their capabilities. Most patients mentioned they are more likely to trust and rely on doctors because doctors are the experts, they already have a relationship with them, or they inherently cannot trust technology (PT17). To an extent, some patients wanted doctors to take responsibility for AI and decide whether and when AI should be used.

A few professionals agreed that doctors’ responsibility entails making these complex decisions regarding AI use. The professionals’ views come from an underlying assumption that AI is a support tool and that SDM can come after the AI (diagnosis or treatment) support (AE13 and AE12). Reliance on doctors is an acceptable and expected behavior for both groups. However, it is also possible this behavior is not an active decision but a consequence of seeing AI as unavoidable, complex, and limiting for patients.

#### Self-Efficacy Through Value Judgments

Most patients judged the value of AI depending on how they saw technology in general or how they assessed AI’s usability for their (current) care plan. Beyond questioning the accuracy or relevancy of AI’s information, patients based these judgments on their personal understandings and views about technology. In this way, patients aimed at preserving their decision self-efficacy often categorically accepting or rejecting AI. Depending on these judgments, their choices would change between wanting and not wanting to be involved in decision-making for AI-supported health care (PT11 and PT6). A few patients, for example, valued their openness to new technologies as positive, which meant they would inherently be inclined to accept AI in all forms. In contrast, other patients categorized themselves as uninterested or mentioned not needing AI at all. Both groups adapted their preferences based on their personal values and what they saw as possible for the way they live their lives. The strategy of patients to judge the value of AI for their own life and decide whether they would like AI to be involved in their care, helps them preserve their self-efficacy and retake the leading role of decision-making regarding AI’s use for their care (PT5). Positively, patients felt capable of making their own choices (especially related to AI-enabled smartwatches). However, there was an ambivalence and polarization between acceptance and refusals. Patients base their value judgments on ideologies of life (eg, whether they are interested in knowing more health information or being informed about health problems) instead of factual information. A few patients reflected on this ambivalence and how to take either position.

## Discussion

### Principal Findings

This paper presents empirical evidence that voices patients' concerns about AI-supported care and challenges to patients’ participation in decision-making. Through interviews with patients and AI professionals, we found that patients are often allocated to a passive standpoint, where they feel unable to participate, a resource to follow their doctor’s recommendations, or make precipitated value judgments about AI’s actual benefits or risks. In that way, this study revealed that external narratives of AI as a force of change have influenced how patients perceive their role and limitations to participate in decision-making. The insights that patients have adapted to limitations by accepting or endorsing a passive role are concerning because patients unknowingly may be giving away their decisional power.

In many ways, the perception of AI as an inevitable and potentially disruptive force in health care reflects both optimism and concerns about its transformative impact. This includes visible uncertainties about how AI systems function, concerns about decision-making authority, and fears of losing personal agency. The themes of this paper, collectively, illustrate the complex and often conflicting ways in which patients navigate the presence of AI in health care settings.

### Comparison With Prior Work

Previous research about patients’ views focused on understanding how they see AI rather than how patients could participate in decision-making in AI-supported care and remain empowered. A scoping review of qualitative research found that stakeholders (eg, doctors and patients) see AI as mostly positive but are cautious about its application [35]. Although the availability of patients’ perspectives in the scoping review was limited, our findings correlate to the sentiments expressed by other patients worldwide and their ambivalent views about AI. Several researchers have found that patients’ positive evaluation of AI depended on the possibility of having oversight by doctors [15-18,35]. For example, patients in Germany had high confidence in their doctors’ abilities to work with AI, but it was unclear if they favored AI or saw AI as an unstoppable development [18]. These findings correspond to our results, in which patients desire their doctors to handle AI and perceive AI as complex and unavoidable. Our research offers further in-depth analysis of how these views are expressed and potentially uncovers how patients and professionals hold these perceptions.

The formation of person-centered care requires that patients are (and feel like) equal partners in decision-making and that their knowledge, wants, and wishes are considered [36]. Therefore, the goal of care shifts from informing patients about diseases to creating an informed space in which patients’ values and preferences are represented and respected. However, sharing information about AI to ensure that patients are informed might be challenging. For example, during our interviews, patients and professionals questioned whether patients could be informed enough to understand AI. Bjerring and Busch [36] state that the inherent complexity of AI systems, especially those with black-box decision layers, is not conducive to person-centered care because of the challenges of understanding and explaining AI. However, theoretical knowledge is not the only possible provision of an informed space. Patients could contextualize their knowledge, recognize the limits of their expertise to seek information from experts, and be empowered enough to decide on a particular action depending on what it means “in their own world” [37]. Therefore, their beliefs and values could affect their decisions more than their factual AI knowledge because it is not expected or attainable that patients are omniscient. As seen in the interviews, patients based their decisions on what they believe and value more than what they know. Understanding how patients expressed and constructed these beliefs and how they affect the desired versus expected participatory level is helpful in supporting person-centered care.

All participants recognized that AI might become a health care presence, leading to social and care changes. Although there was no overall positive or negative outlook regarding these changes, myths and preconceptions of AI informed patients’ perceptions. Previous research has found that myths regarding AI behavior are a common point of discussion in media and science fiction works [38-41]. Therefore, the image of AI is constructed from the underlying hopes and fears of how AI can improve or harm our future [38,39,42]. During the interviews, it was apparent that the myths of a powerful AI that is also complex and overbearing informed our interviewees' hopes and fears, in particular for patients. These perceptions are a potential driver of the participatory limitations patients and professionals perceive. For example, it is challenging to preserve self-determination if something is an unavoidable self-fulfilling prophecy. Although hopes and fears could be detached from the current reality of technology, they influence how patients envision their interactions with AI and what patients see as possible. Debunking myths and communicating with patients could be a tool to overcome preconceptions and indirectly encourage them to consider participation in decision-making in AI-supported care. Media and AI professionals may need to counteract these perceptions and communicate with patients about AI in a balanced way.

Patients might be willing to accept soft forms of paternalism where doctors handle AI and involve patients after the initial AI decision support. However, previous reports found that patient-controlled or SDM was perceived as more satisfactory and higher quality, even if a patient preferred a passive role [43]. It is possible that patients are undergoing a process of adaptive preference formation where they are navigating the changes AI brings by adapting in ways they see as possible and feasible. Patients might adjust their preferences (passive vs active role) due to the lack of self-trust, as they might see their values, ideas, and fears as unimportant, hampering their ability to make authentic decisions [27,44]. However, it remains unclear whether these adaptations are because of external influences such as social expectations or media narratives or if they express their true wishes and follow their personal values. For example, when patients believe AI is beyond their capabilities, they may feel their only option is to hand over the responsibility for AI to others. If professionals endorse the narratives that originated these preferences (eg, patients cannot handle AI), they might legitimize and reinforce something potentially inappropriate or inauthentic for patients. Most patients accept or reject AI “as is” without considering how or if to change AI for their (own) patient advantage. For some scholars, adaptive preferences are inherently nonautonomous as these are only a response to the limitations present in the context [27,44]. Therefore, endorsing these adaptive preferences could jeopardize patients’ autonomy in decision-making regarding AI-supported health care and ultimately be an impediment to person-centered care.

When the goal is to encourage patients to participate in decision-making, the insights of our research request to reconsider patient education to support knowledge acquisition, judgment skills, and empowerment aspects simultaneously. Therefore, it requires emotional, behavioral, and educational components [37,45]. Patients must be made aware of their rights and the possibilities they have to decide whether and how to use AI. Moreover, education should clarify that their participatory decisions could affect their satisfaction or what are acceptable practices with AI. The aim is to demystify AI, provide usable knowledge, and support patients' self-assessment of competence and abilities [46]. In the age of AI, institutions and professionals are required to guide patients’ learning while still respecting and encouraging their health deliberations [46]. Professionals should stop narratives of unavoidable AI and patients’ loss of capabilities. Instead, professionals must promote a view where patients’ unique expertise in their symptoms, experiences, and health goals are valuable insights for decision-making. Policy makers might also need to promote person-centered AI by explicitly stating patients' rights and their options for decision-making in AI-supported care. One caveat is that empowerment and participation should not be mistaken for taking an active role as this is not the only option, but rather should include and respect all patient choices ranging from active to passive. The goal is to prevent patients from adapting their preferences due to perceived limitations and encourage them to decide how to participate in decision-making in AI-supported care.

### Limitations

One limitation to consider regarding the quality of the interview data relates to the nature of the subject. AI is used as a general term during the interviews to facilitate patient conversation and avoid technical jargon. For our context, the term AI refers to ML and its black-box subtypes, as reflected in the phrasing of the interview questions and vignettes. However, AI is a general descriptor that can be interpreted differently. A few patients and professionals mentioned difficulties involved in using the concept. For example, PT9.2 expressed about AI, “I think the word is wrong or the phrase. Because do you want something artificial or do you want something real? (...) So the term is challenging.” Using the term AI could have shaped interviewees' answers because of the fears and preconceptions associated with the term, which may not reflect specific health care concerns. However, AI is commonly used in media communication, and all patients have already been exposed to this term. While this issue was mitigated to some extent by providing vignettes with scenarios of CDSS, it was impossible to completely eliminate the risk of misinterpretations or misconceptions. Differences in language between German/Swiss German and English could also have influenced how patients interpreted and answered the questions. This research was conducted prior to the release of large language models like ChatGPT, resulting in lower general awareness of common AI behaviors and capabilities—a potential limitation of the study.

The recruitment method meant that only those patients with time and interest might have participated in our study; also, the patient sample of this study skewed towards men and older people. AI views and the envisioned patient role may be influenced by gender or cohort. For example, men might have more favorable attitudes toward AI, and older people might have less health and AI literacy, which could affect their levels of self-trust [47]. One couple was interviewed together, which may have introduced biases in their responses. Their presence together could have influenced each other's answers, either consciously or unconsciously, or increased the likelihood of providing socially desirable responses [48]. However, paired-depth interviewing also offers the possibility for dynamic discussions and deeper insight into shared perceptions as observed in our participants. Therefore, the results of this paper should be interpreted with consideration of both its strengths and limitations. The core knowledge of patients about AI was not measured during our interview, which could affect how they answered the questions. Given the qualitative methodology, our findings cannot be extended to a broader population and must be considered in context.

### Conclusions

The ideas and preconceptions of patients and professionals about AI have influenced how they see the different possibilities within the patient role. External narratives of AI as a force of change and the fears associated with its potential harms have caused patients to internalize limitations regarding their role and capabilities to handle it. Patients have aimed to navigate AI’s changes by adapting their preferences to either rely on those considered experts or by deciding to accept or refuse AI categorically. However, both adaptive strategies carry a potential risk of disempowerment and passivity because patients feel they cannot do much to stop, change, or improve the course of AI in health care. If the goal is to empower patients to be active partners, these adaptive responses might be insufficient to position them at the center of their care. Patients' empowerment and involvement in AI decision-making might be better understood as a dynamic process of balancing expectations and preferences and the realities of the technology and clinical practice. Professionals, institutions, and policy makers must support that patients realize they can have the possibility to help shape AI for health care. The goal is to put patients in a position in which power is manifested not by the ability to do but by the ability to decide how to do it.

## Appendix

Multimedia Appendix 1 Interview guides.

Multimedia Appendix 2 Sample characteristics.

## Abbreviations

AE: AI professional participant
AI: artificial intelligence
CDSS: clinical decision support systems
EXPLaiN: Explainability of Digital Transformation and Data Technologies Using Artificial Intelligence
ML: machine learning
PT: patient participant
RTA: reflexive thematic analysis
SDM: shared decision-making
SRQR: standards for reporting qualitative research

## Data Availability

The datasets generated and analyzed during this study are available from the corresponding author on reasonable request.
