# Supplementary material for: Exploring Patient Participation in AI-Supported Health Care: Qualitative Study
Source: JMIR AI. 2025 May 5;4:e50781. doi: 10.2196/50781 (PMC12089863; doi:10.2196/50781)
Supplement: Multimedia Appendix 1 [file ai_v4i1e50781_app1.pdf]

MULTIMEDIA_APPENDIX_1: INTERVIEW GUIDES

A) PATIENTS INTERVIEW (GERMAN VERSION)

Einleitende Fragen

#	Fragen	Folgefragen
1	Können Sie mir ein wenig über sich erzählen?	• Was ist der Grund für Ihren Besuch beim Kardiologen?
2	Benutzen Sie Smartwatches (tragbares Gerät)?	• Falls ja:<br>○ Warum benutzen Sie Wearables?<br>○ Welches Wearable benutzen Sie?<br>○ Wie gefällt es Ihnen?<br>• Wenn nein:<br>○ Warum benutzen Sie keine Wearables?<br>○ Sind Sie daran interessiert, eines zu verwenden?

Vignette (Inspiration von https://arxiv.org/pdf/1905.05134.pdf Seite 4 und Anhang A)

Ich möchte Ihnen jetzt eine Geschichte über eine Frau/einen Mann erzählen.

Vignette a (Thema: allgemeine Ansichten und Bedenken):

- Eine Person besitzt eine Smartwatch (mit KI), die die Herzfunktion messen kann (Herzfrequenz, Atemfrequenz, Messung des Sauerstoffgehalts im Blut usw.).

- Eines Tages sagt die Smartwatch zu dieser Person, dass nach der Analyse der Daten etwas mit ihrem/seinem Herzen nicht gut ist.

1) Was haltet ihr von dieser Smartwatches (KI)?
2) Haben Sie Bedenken beim Einsatz von Smartwatches (KI)?
3) Was würden Sie mit der Popup-Nachricht tun?
4) Was halten Sie davon, dieser Technologie (KI, Smartwatches) zu vertrauen?

Vignette b (Thema: Arzt-Patienten-Beziehung und Erklärbarkeit):

Jetzt haben wir eine andere Situation. Es gibt einen Arzt, der KI verwendet. Diese KI wird im Hintergrund verwendet. Diese KI unterstützt den Arzt bei der Entscheidungsfindung entweder für Diagnose oder Behandlung.

5) Welche Fragen würden Sie Ihrem Arzt stellen?
6) Möchten Sie wissen, dass der Arzt KI verwendet? Warum?
7) Möchten Sie, dass der Arzt Ihnen erklärt, wie die KI funktioniert?
8) Möchten Sie verstehen, wie die KI funktioniert?
9) Wenn die KI Ihre Diagnose stellen würde, wie würden Sie sich fühlen?
10) Glauben Sie, dass Ärzte die KI verstehen sollten?

Vignette c (Thema: Patienten Risiken Vorurteilen und Andere Meinung):

11) Es besteht die Möglichkeit, dass die KI manchmal Vorurteile hat. Was denken Sie über dieses Risiko?
12) Manchmal können der Arzt und die KI anderer Meinung sein. Was würden Sie in dieser Situation tun?

Abschlussfragen

11	Was können Patienten Ihrer Meinung nach gegen den Einsatz von KI in der Klinik tun?
12	Wie sollte die Regierung Ihrer Meinung nach vorgehen, um KI im Gesundheitswesen einzusetzen?
13	Gibt es noch etwas, das Ihrer Meinung nach beim Einsatz von KI wichtig ist?

B) AI PROFESSIONALS INTERVIEW

Interview questions

a) Introductory questions

#	Questions	Follow up probes
1	Can you tell me a little bit about yourself?	● What is your background?<br>● Where are you located?<br>● What is your role?<br>● Can you tell me more about your current projects<br>● Do you work with ML/AI?<br>● How did you become interested in ML/AI?<br>● What is your experience with ML?

b) General questions about using AI in clinical practice

#	Questions	Follow up probes
2	I would like to start discussing using AI/ML in clinical practice.<br>What do you think about using ML in healthcare?	● In which cases do you think is useful and NOT useful?<br>● Has it been beneficial so far?
3	How/Where do you think ML can/should be implemented in clinical practice?	● Is this a high priority need in clinical practice?<br>○ Is there any specific application that you think would be the most useful?
4	What do you think about using ML for supporting doctors and patients in clinical decisions?	● Do you have any concerns?<br>● Do you see any benefits?<br>● Could it affect<br>○ Doctor-patient relationship?<br>○ Trust?<br>○ Autonomy?<br>○ Informed consent?<br>● Would understanding ML be relevant in this context?<br>○ What type of knowledge?<br>● How would you define explainability?

5	What would you consider the biggest challenges of using ML in healthcare ?	● What are the biggest obstacles to overcome?<br>● What challenges have you faced using ML in clinical practice?<br>● Do you have any ethical or regulatory concerns?<br>● Do you think ML can cause concerns for physicians?<br>● Do you think ML can raise concerns for patients?
6	What would you consider the biggest challenges of using ML for supporting doctor and patients in clinical decisions?	● How do you think policy makers should be involved?<br>● How do you think clinicians should be involved?<br>● How do you think computer scientists should be involved?<br>● How do you think ethicists should be involved?<br>● How do you think patients should be involved?
7	I would like to know how you think we could advance the implementation of ML for the analysis of health data?	● How do you think policy makers should be involved?<br>● How do you think clinicians should be involved?<br>● How do you think computer scientists should be involved?<br>● How do you think ethicists should be involved?<br>● How do you think patients should be involved?
8	Which regulatory aspects are important for the implementation of ML in healthcare?	● Which regulations do you think are important?<br>○ Would you prefer a strong regulatory framework, where different usages and situations are defined and there is a quite clear procedure, or a soft regulatory framework where general usages are defined but the particular practice can still be rather discretional?<br>● Do you know any regulatory frameworks that are applied?<br>● Which aspects need a lot of consideration?

		● Are there any major concerns that should be addressed by regulations?

c) VIGNETTES

Cardiology cases (comparison if patients' basal risk change answers - context related questions):

If the interviewee has mentioned another case. Ask them to expand on it and ask the same questions/probes related to their example.

Let's consider a fictional scenario where someone owns a smartwatch. This smartwatch uses artificial intelligence to check the functioning of the heart (like heart rate, respiration rate, saturation, …).
Scenario 1: Jane is 40 years, has no previous diseases, feels healthy.
Scenario 2: Max is 70 years, lives with hypertension and diabetes and feels healthy.
One day there is a pop-up message saying that they have a change in the rhythm of their heart (cardiac arrhythmia - atrial fibrillation) .

Additional medical detail (if needed):
Jane has no family history of cardiac disease. Jane has no symptoms. Previous visit to the doctor all results were in normal standards.
Max takes medication for his hypertension and diabetes and has already some signs in previous visits of deteriorating renal function.
The smartwatch that they use has been validated to take ECG (single-lead) to diagnose atrial fibrillation.

9	How do you think these patients should react to the pop-up message?	● Do you think that they should believe the pop-up message? What are your thoughts on trust?<br>● What criteria are important for you to trust the results?<br>● Who should trust the results?<br>● How important is this concept for

		you? Why?<br>● What information or facts would you need to evaluate the suggested diagnosis?<br>● Should Jane and Max visit the doctor? Emergency or request a normal consultation?
10	If Max/Jane decides to book an appointment with the doctor, what do you think they should say to their doctor?	● How should they share the data of the smartwatch with the doctors?<br>● Should they mention that the reason for consultation is the smartwatch pop-up message?<br>● How do you think Max/Jane feel about using technology to provide them more information about their health and have the capacity to say this to their doctor?

Vignette to compare other case where the clinical decision is based only on information (comparison of positions between patients and doctors and if type of disease change answers- context related questions):

Now we have a patient named Ruth, 67 years old and she is feeling dizzy. She decides to go to the clinic for a check-up appointment.

The hospital she visits is implementing ML technology and the doctor will be using it during the consultation to support the diagnosis. During the check-up, the doctor adds all the symptoms. The doctor mentions that there is a chance of diabetes and that she is at risk for complications.

Additional medical detail (if needed):

During the medical interview, Ruth mentions the triad of diabetes (thirst, polyuria and increased appetite). Ruth's blood sugar levels are high, also her haemoglobin A1C is high.

11	How do you think the doctor should communicate the usage of ML?	- Do you think that the doctor should mention the use of ML to support the diagnosis? - What should the doctor disclose regarding the usage of ML? - How should the doctor explain ML to Ruth? - What other information should the doctor ask, mention or share with Ruth?
12	What do you think about Ruth's consent to use ML?	- Do you think consent is necessary? When? - What do you think about using her data? Do you have any concerns about health data handling? - If the context of consent changes and the ML would be suggesting an invasive procedure (e.g. surgery to remove the appendices) would that change any of your previous answers?
13	How would you feel about the doctor using ML to support the diagnosis?	- Would it be necessary for the doctor to understand ML? To what degree? - Would it be necessary for the patient to understand ML? To what degree? - What does explainability mean to you? - How would you evaluate explainability? - How important is this concept for you? Why? *Additional medical probes:*

		- *How would you handle a disagreement between your clinical judgement and the machine's suggestions?*<br>- *How do you feel if the context of this case will be in an emergency situation? Would that change any of your previous answers?*<br>- *How do you think ML should handle medical uncertainty?*<br>- *Would you feel supported by the usage of ML?*
14	Do you think using ML during the consultation will affect in any way Ruth's relationship with her doctor?	• How do you think patients will react to the knowledge of doctors using technology to support their decisions?<br>• What would be the advantages and disadvantages to the doctor-patient relationship of using ML during the consultation?<br>• *Additional medical probes:*<br>- *How would you feel about telling patients you are receiving ML support to make clinical decisions?*

-

Vignette private-public relationship

Let's consider the two scenarios discussed and add that in both cases, the ML/AI used to analyse the data was a private company. For example, Apple would be the one analysing the data with the apple watch.

15	What would be your opinion regarding the involvement of private companies?	• What do you think about sharing data from public hospitals with private companies?<br>• Do you have any ethical or legal concerns regarding their involvement?

d) Closing questions

16	Are there any other practical, medical, and ethical issues that you think are important for the scenario that we have not discussed?	• Is there some concern that you have that was not addressed?<br>• Do you have any recommendations?
