# Supplementary material for: Exploring Patient Participation in AI-Supported Health Care: Qualitative Study
Source: JMIR AI. 2025 May 5;4:e50781. doi: 10.2196/50781 (PMC12089863; doi:10.2196/50781)
Supplement: Multimedia Appendix 2 [file ai_v4i1e50781_app2.pdf]

MULTIMEDIA_APPENDIX_2: SAMPLE CHARACTERISTICS

a)Summary of sample and interview

Subgroup		Patients (n=21)	AI professionals (n=21)
**Sample characteristics**	Background	Diverse educational backgrounds (from technical education to university degree holders)	Professionals from medicine, sociology, law, philosophy, ethics, bioinformatics, or computer science
	Sex	7 women, 14 men	6 women, 15 men
	Location	21 patients were recruited in Basel, Switzerland	13 professionals from Switzerland and 8 from Germany
**Interview characteristics**	Duration	Average approx. 23 minutes (12–40 minutes)	Average approx. 40 minutes (30 minutes to 1 hour)
	Language	16 interviews were conducted in Swiss German or standard German. 5 interviews were conducted in English	21 interviews conducted in English

b)Sample characteristics of AI professionals

AI Professional (Rn)	Background	Location	Gender
1	Medicine	Switzerland	M
2	Medicine/AI	Switzerland	M
3	Bioethics/Medicine	Switzerland	W
4	Law	Switzerland	M

5	Data	Switzerland	W
6	Bioethics	Germany	M
7	Law	Switzerland	W
8	Policy/AI	Germany	M
9	Bioethics	Germany	M
10	Law	Switzerland	M
11	Medicine	Switzerland	M
12	Law	Switzerland	M
13	AI/Biology	Germany	M
14	Medicine/Data	Switzerland	M
15	Computer Science/AI	Switzerland	M
16	Medicine/AI	Switzerland	M
17	Medicine/AI	Germany	W
18	Medicine/AI	Germany	W
19	Medicine	Switzerland	M
20	AI	Germany	W
21	Medicine/AI	Germany	M

c)Sample Characteristics of Patients

Patient No.	Language	Profession	Level of Education	Gender
Rn1	Swiss German	Technology	University	M
Rn2	Swiss German	Retired	University	M
Rn3	French	Books curator/antiques	University	M
Rn4	German (Swiss)	Retired (previous work in tech)	University	W
Rn5	German (swiss?)	Technology	University	W
Rn6	Swiss German	Retired	Unknown	M
Rn7	Korean	Art	University	W
Rn8	German	(unknown)	High School	M
Rn9 (pt 1)	English	Pharmaceutical	University	M

Rn9 (pt 2)	English	Pharmaceutical	University	M
Rn10	Swiss German	Doctor	University	M
RN11	Swiss German	unknown	unknown	M
RN12	German	retired	unknown	W
RN13	Swiss German	Pharmaceutical	University	M
RN14	Swiss German	Postal worker	Apprenticeship	W
RN15	Swiss German	Retired (previous work in social work)	Apprenticeship	M
RN16	German	Nurse	University	W
RN17	Swiss German	Retired	University	W
RN18	Swiss German	unknown	Apprenticeship	M
RN19	Swiss German	Federal government employee	Apprenticeship	M
RN20	Swiss German	Doctor	University	M
